# Comparison of Kidney Transplantation Outcomes Between Patients with and Without Pre-transplantation Bariatric Surgery: a Systematic Review

**DOI:** 10.1007/s11695-022-06308-1

**Published:** 2022-10-13

**Authors:** Pouria Mousapour, Jonathan Ling, Edward Zimbudzi

**Affiliations:** 1 Obesity Research Center, Research Institute for Endocrine Sciences, Shahid Beheshti University of Medical Sciences, Tehran, Iran; 2 Department of Nephrology, Monash Health, Melbourne, VIC 3168 Australia; 3 School of Public Health and Preventive Medicine, Monash University, Victoria, 3004 Australia

**Keywords:** Kidney transplantation, Severe obesity, Bariatric surgery, Systematic review

## Abstract

This systematic review evaluated the impact of bariatric surgery, performed to improve eligibility for kidney transplantation, on post-transplantation outcomes. A systematic literature search was performed for articles published by 30 January 2022. A total of 31 studies were included. Among patients without pre-transplantation bariatric surgery, 18 studies reported 13.7% graft loss and 9.1% mortality within 5 years’ post-transplantation. Among recipients with pre-transplantation bariatric surgery, 15 studies reported 8.7% graft loss and 2.8% mortality within 1 month to over 5 years’ post-transplantation. Two case–control studies comparing post-transplantation outcomes between recipients with and without prior bariatric surgery demonstrated no significant differences between groups for graft loss, patient mortality, delayed graft function, wound complications and lymphocele. Non-randomized selection of patients and different lengths of follow-up limit the results of this study.

**Supplementary Information:**

The online version contains supplementary material available at 10.1007/s11695-022-06308-1.

## Introduction

Obesity, the state of excessive adipose tissue accumulation, is associated with multiple comorbidities such as diabetes, hypertension and atherosclerosis and is an independent risk factor for chronic kidney disease (CKD) [1, 2]. Severe obesity, defined as body mass index (BMI) ≥ 35 kg/m^2^, has emerged as a public health challenge. In 2014, the global prevalence of severe obesity was 2.3% in men and 5.0% in women had, and it was forecasted that 6% of men and 9% of women will have been severely obese by 2025 worldwide [3]. Compared to the healthy weight population (BMI 18.5–24.9 kg/m^2^), individuals with severe obesity are 1.5 times more at risk of premature all-cause mortality [4, 5]. Concurrently, from 1990 to 2017, the global prevalence of CKD has also increased by 30% from 1990, reaching 700 million cases worldwide; and in parallel with increasing availability of renal replacement therapy over this time period, the global incidence end stage kidney disease (ESKD) treated by dialysis and kidney transplantation increased by 43.1% and 34.4%, respectively [6].

Kidney transplantation remains the definitive therapy for patients with ESKD [7]; however, obesity remains a risk factor for kidney transplantation, as many institutions still consider BMI ≥ 40 kg/m^2^ as an absolute contraindication and BMI 35–39.9 kg/m^2^ as a relative contraindication to kidney transplantation [8]. Despite successful outcomes, kidney transplantation in obese recipients is reportedly associated with increased wound complications, acute rejection and delayed graft function as well as lower graft loss and patient mortality rates within 3 years post-transplant, in comparison with their non-obese counterparts [9]. In order to minimize such complications, pre-transplantation weight reduction for obese candidates is usually advised [9].

Bariatric surgery is recognized as the most rapid, most effective and sustainable treatment of severe obesity and its comorbidities, including type 2 diabetes mellitus, hypertension and dyslipidemia, as compared to other weight loss strategies [10, 11]. A meta-analysis containing studies with over 10 years of follow-up data on weight loss after bariatric surgery demonstrated excess weight loss percentage (EWL%) to be 56.7% after gastric bypass, 58.3% after sleeve gastrectomy and 45.9% after adjustable gastric banding [12].

Bariatric surgery improves eligibility for kidney transplantation in candidates with severe obesity [13]. Besides, patients with severe obesity and ESKD have an increased all-cause mortality risk, that is shown to decrease following bariatric surgery [13], but this may be at the cost of slight increases in the risks of infections, cardiovascular events and graft dysfunction [14, 15]. Hence, it is unclear whether the potential benefits of pre-transplantation weight loss via bariatric surgery outweigh the surgical risks in renal transplant candidates with severe obesity.

Although a number of studies have shown that bariatric surgery in patients with severe obesity improves access to kidney transplantation following significant weight loss, a review of the literature assessing post-transplantation outcomes in these patients was lacking. Therefore, we aimed to systematically review post-transplantation outcomes between morbidly obese patients with and without pre-kidney transplantation bariatric surgery.

## Methods

This systematic review was guided by the Cochrane Handbook for Systematic Reviews of Interventions [16] and conforms to the reporting guidelines of the Preferred Reporting Items for Systematic Reviews and Meta-Analyses (PRISMA) statement recommendations [17]. The systematic review protocol was registered on PROSPERO (registration number CRD42021224801).

### Inclusion and Exclusion Criteria

The Population, Intervention, Comparison, and Outcome (PICO) framework was used (Supplementary Table 1), to formulate the inclusion and exclusion criteria.

### Cohort

Kidney recipient candidates aged 18 years or above with severe obesity undergoing bariatric surgery pre-transplantation were included. Patients who had bariatric surgery after or at time of kidney transplantation were excluded.

### Interventions

Bariatric procedures such as adjustable gastric banding, sleeve gastrectomy, or gastric bypass performed prior to kidney transplantation were considered. Studies including dietary or medical interventions for pre-transplantation weight loss were excluded.

### Comparator

Patients with severe obesity who underwent kidney transplantation without any documented attempts at weight loss were included. We excluded multi-organ transplant recipients.

### Outcomes

#### Primary

Kidney graft loss and patient mortality.

#### Secondary

Acute rejection, delayed graft function and post-transplantation complications including systemic infections (e.g. pneumonia, urinary tract infection and cytomegalovirus reactivation), wound dehiscence/infection and lymphocele.

Any study reporting either primary or secondary outcomes was included, and those without post-transplantation follow-up were excluded. We limited the outcomes of interest to 5 years of follow-up in order to minimize information bias.

### Search Strategy

The Excerpta medica (EMBASE), PubMed and Scopus were searched from inception up to 30 January 2022 for studies meeting previously stated inclusion criteria. We only included papers written in English, due to limited translation resources. The following search terms were used: ‘bariatric surgery’/exp, ‘metabolic surgery’/exp, ‘gastric banding’/exp, ‘sleeve gastrectomy’/exp, ‘gastroplasty’/exp, ‘gastric bypass’/exp, ‘morbid* obes*’, ‘severe* obes*’, ‘renal transplant*’ and ‘kidney transplant*’ (Supplementary Table 2).

Title and abstract screening as well as full-text reviews were performed by two independent reviewers (PM and EZ). Any disagreements were resolved via a third reviewer (JL). Additionally, reference lists from included texts were reviewed for studies suitable for inclusion. Whenever further clarification was required, corresponding authors of the eligible studies were contacted.

### Data Extraction and Critical Appraisal

Two reviewers (PM and EZ) independently extracted data. The collected information included first author’s name, year of publication, country, study design, number of participants, mean age, sex proportion, duration of follow-up and the outcomes of interest following kidney transplantation.

The methodological quality of the included studies was assessed independently by two authors (PM and EZ) using the Newcastle–Ottawa scale (NOS) for evaluating the methodological quality of cohort and case–control studies [18] and a modified version of the NOS designed for case reports and case series [19]. The NOS quality assessment tool rates studies in three domains: the selection of the study groups, the comparability of the groups and the ascertainment of the outcome of interest to a maximum of eight stars (four stars for selection, one for comparability and three for exposure domains, respectively). Good study quality was defined as three or four stars in selection domain AND one star in comparability domain AND two or three stars in exposure domain; fair quality was defined as two stars in selection domain AND one or two stars in comparability domain AND two or three stars in exposure domain; and poor quality was defined as no or one star in selection domain OR no stars in comparability domain OR no or one stars in exposure domain. The modified version of NOS uses four domains (election, ascertainment, causality and reporting) to rate each study with a maximum of eight stars; one for the selection domain, two for ascertainment, four for causality and one for reporting. Good quality was defined as at least one star in each domain; fair quality was defined as no stars in selection domain, but all other sections starred; and poor quality was defined as no stars in any of the ascertainment, causality or reporting domains. Since the modified version of the NOS was originally adapted for the assessment of drug reactions, a couple of questions in the Causality section were not applicable to our study as they were related to dosage. Any disagreement between the reviewers was resolved via the third reviewer (JL).

### Data Analysis

Post-kidney transplantation outcomes were grouped according to whether the patient(s) had undergone pre-transplantation bariatric surgery. If more than two randomized clinical trials had derived risk indices for similar outcomes, we aimed to aggregate the data via a meta-analysis Unfortunately, only two non-randomized, retrospective studies were identified via our search, with the remaining studies being case reports and case series; thus, we elected to descriptively analyze our primary outcomes by reported patients’ characteristics including recipients’ age and gender, type of donor (living versus cadaveric), recipients’ BMI at transplantation and type of bariatric surgery. These characteristics were chosen due to their role as potential confounders of our primary and secondary post-kidney transplantation outcomes.

## Results

**Fig. 1:**
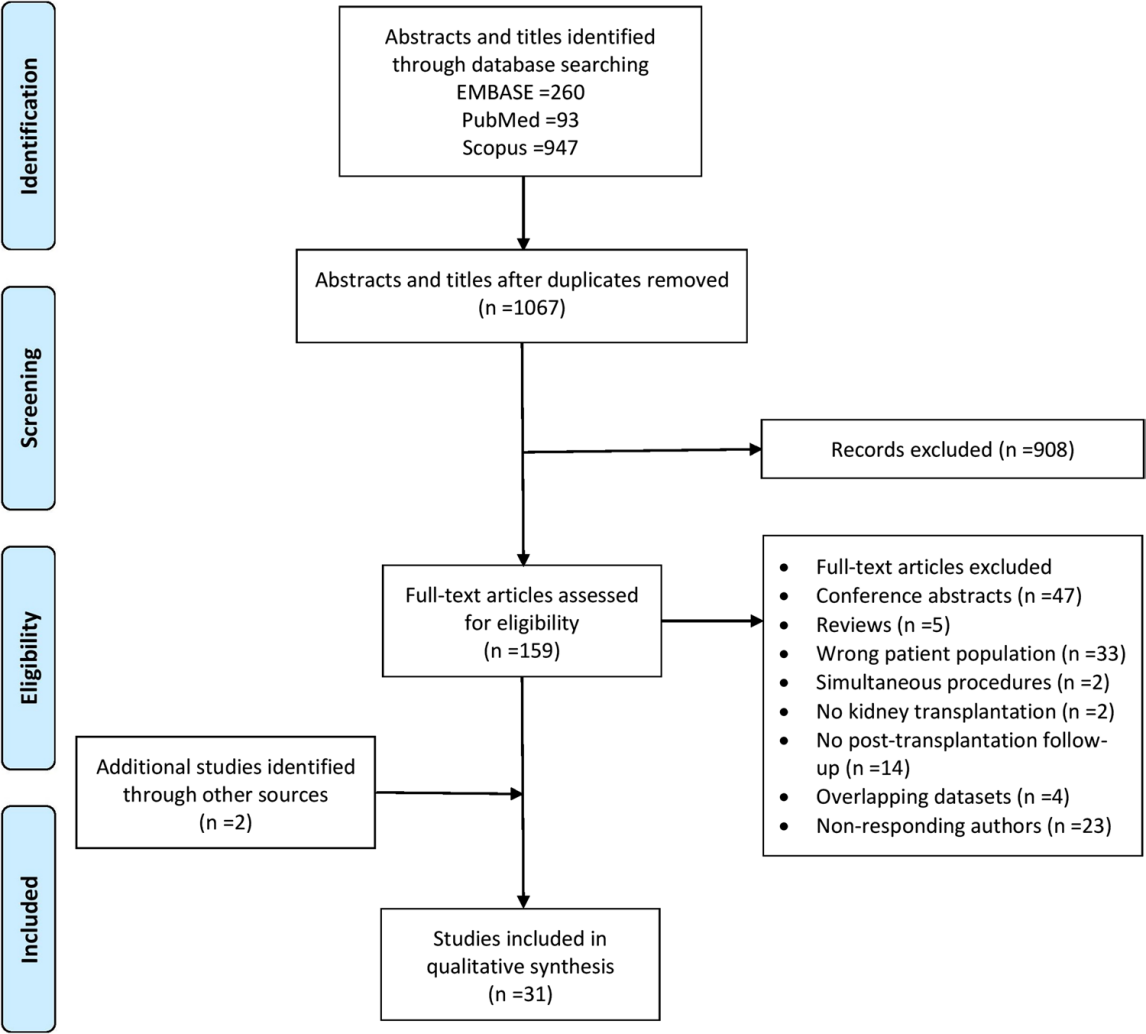
PRISMA flow diagram showing how studies were screened

The results of the systematic search are shown in Fig. 1. The literature search identified 1300 references. After excluding 233 duplicates, 1067 titles and abstracts were screened for eligibility, from which 908 were excluded. A total of 159 articles were retrieved for full-text review. Forty-seven conference abstracts, five reviews and 55 studies were excluded due to not meeting the inclusion criteria and overlapping cohorts with included studies [20–23]. Out of the remaining 52, corresponding authors of 24 studies were contacted for data on patients who had not been sub-grouped as severe obesity; however, only one author responded, and accordingly, 23 studies had to be excluded. At this stage, two studies were added from hand-searching of a systematic review’s reference list [24]. Finally, 31 studies were reviewed.

### Study Characteristics and Quality Assessment

Study characteristics for both groups (obese patients undergoing bariatric surgery pre-kidney transplantation and obese patients without bariatric surgery pre-kidney transplantation) are detailed in Table 1 and Table 2. The study quality assessment using NOS for the two case–control studies and a modified version of NOS for case reports and case series is detailed in Table 3.

**Table 1:** Studies and characteristics of end-stage kidney disease patients with morbid obesity undergoing kidney transplantation without prior bariatric surgery

Authors, year	Study design	Country	Patients, *n*	Sex, *n* (Male/female)	Age, yr	Donor type, *n* (Living/Cadaver)	Follow-up, month	Acute rejection	Delayed graft function	Graft loss	Complications, *n* (cause)	Mortality, *n* (cause)
Marks et al. [25], 2004	RCoS	USA	23	7/16	Mean 44.9	11/12	Mean 36	0	0	3	7 (Wound infection)	1 (Bleeding) 1 (acute post-transplant thrombosis) 1 (Graft failure)
Cacciola et al. [26], 2008	RCoS	UK	24	12/12	45 (Range 20–61)	0/24	Mean 60	3	NR	9	5 (Wound infection)	2 (Cardiac) 3 (Sepsis) 2 (Graft failure) 2 (Others)
Olarte et al. [27], 2009	RCoS	USA	43	NR	NR	NR	Mean 12	NR	NR	NR	4 (Pneumonia) 2 (UTI) 2 (Wound infection)	0
Bennett et al. [20], 2011	RCoS	USA	94	40/54	Median 51	32/62	Range 12–105.6	NR	30	12	15 (Wound infection)	4 (Cardiovascular) 3 (Sepsis) 1 (Malignancy) 2 (Unknown)
Ditonno et al. [28], 2011	PCoS	Italy	10	NR	Mean 48 (Range 39–55)	NR	Mean 53 [range 3–112]	2	4	0	6 (Lymphocele) 2 (Wound infection) 2 (Infection)	2 (Sepsis)
Karabicak et al. [29], 2011	RCoS	USA	61	35/26	Mean 47.6 ± 15.8	15/46	Mean 92.8 ± 2.1	9	11	16	NR	9 (NR)
Curran et al. [30], 2014	PCoS	Canada	74	35/39	Mean 50.8 ± 10.4	35/39	Mean 60	27	NR	27	NR	NR
Garcia-Roca et al. [31], 2017	RCoS	USA	612	230/382	Mean 47.9	612/0	Mean 36	12	33	38	NR	15 (Sepsis) 12 (Cardiovascular) 2 (Cerebrovascular) 1 (Bleeding) 1 (Malignancy)
Tremblay et al. [32], 2016	RCoS	USA	57	31/26	Mean 47.9 ± 11.9	43/14	Mean 41 ± 29	NR	4	8	NR	NR
Schachtner et al. [33], 2017	RCoS	Germany	21	9/3	Mean 52 (Range 28–78)	3/18	Median 70 (range 0–128)	6	7	3	4 (Sepsis) 6 (Wound infection)	2 (Sepsis) 1 (Cardiovascular) 1 (Malignancy) 1 (Others)
Liese et al. [34], 2018	RCoS	Germany	15	4/11	Mean 53.6 ± 8.9	2/13	Range 36–96	1	6	3	6 (Wound infection)	NR
Veasey et al. [35], 2018	RCoS	USA	105	65/40	Mean 52	97/8	Mean 6	NR	36	19	1 (UTI) 2 (Ureteral leak) 8 (Wound infection) 5 (Lymphocele) 1 (Pneumonia)	6 (NR)
Thomas et al. [36], 2018	Case–control	USA	19	10/9	Median 37.8 [ Range 36.0–42.0]	0/19	Median 72 [range 48–72]	3	4	5	5 (Pneumonia) 6 (UTI) 3 (CMV reactivation) 1 (Wound dehiscence) 5 (Lymphocele)	1 (Cardiovascular) 1 (Sepsis) 1 (Pneumonia)
Kostakis et al. [37], 2019	RCoS	UK	807	421/386	Mean 48.9 ± 11.7	570/237	Mean 120	NR	182	5-year: 113 10-year: 250	NR	5-year: 81 (NR) 10-year: 210 (NR)
Bellini et al. [38], 2020	RCoS	UK	20	13/7	Mean 50.6	3/17	Mean 36	13	2	2	NR	0
Outmani et al. [39], 2020	Case–control	the Netherlands	133	64/69	Median 53.1 (IQ 40.9–63.3)	105/28	Median 61	NR	37	27	42 (UTI) 10 (Wound infection) 5 (Wound dehiscence) 5 (Lymphocele) 3 (Pneumonia)	6 (Sepsis) 6 (Cardiovascular) 4 (Malignancy) 1 (Graft failure) 6 (Others)
Prudhomme et al. [40], 2020	RCoS	Europe	7	5/2	Mean 53.3 ± 13.4	7/0	Mean 3 (range 1–4)	1	1	0	1 (Pneumonia)	0
Spaggiari et al. [41], 2020	RCT*	USA	9	7/2	Mean 45.4 ± 10.5	9/0	Mean 36	1	0	2	2 (Wound infection) 1 (UTI)	0

*RCoS*, retrospective cohort study; *NR*, not reported; *UTI*, urinary tract infection; *CMV*, cytomegalovirus

^*^Only data from one group were included

**Table 2:** Studies and characteristics of end-stage kidney disease patients with morbid obesity undergoing bariatric surgery prior to kidney transplantation

Authors, year	Study design	Country	Total patients, *n*	Transplanted patients (Male/Female), n	Age, yr	Type of bariatric procedure	Donor type, *n* (Living/Cadaver)	Follow-up, month	BMI at transplantation, kg/m^2^	Acute rejection	Delayed graft function	Graft loss	Complications, *n* (cause)	Mortality, *n* (cause)
Newcombe et al. [42], 2005	CR	Australia	2	1 (1/0)	65	AGB	NR	NR	33.2	0	0	0	NR	0
Buch et al. [43], 2006	CR	United States	1	1 (0/1)	59	AGB	0/1	1	NR	0	0	0	NR	0
Alexander et al. [44], 2007	CS	United States	41	9 (NR)	NR	RYGB	NR	Mean 60	NR	NR	NR	NR	NR	0
Koshy et al. [45], 2008	CR	Australia	3	3 (2/1)	Range: 27–49	AGB	2/1	Range 6–24	Range 30.4–37	0	0	0	NR	0
Marszalek et al. [46], 2012	CR	Poland	1	1 (0/1)	55	AGB	0/1	NR	29	0	1	0	NR	0
Al Bahri et al. [47], 2017	CS	United States	4	4 (1/3)	Range [43–60]	RYGB	1/3	NR	NR	0	0	0	NR	0
Kienzl-Wagner et al. [23], 2017	CS	Austria	8	7 (3/4)	Range [25–62]	SG	0/7	Range 5.6–45.6	Range 28.1–35.7	0	2	0	1 (Lymphocele) 3 (UTI) 1 (CMV reactivation) 2 (Wound dehiscence)	0
Al Sabah and Al Haddad [48], 2017	CR	Kuwait	1	1 (1/0)	52	SG	0/1	42	31.5	0	0	0	0	0
Thomas et al. [36], 2018	Case–control	United States	31	14 (5/9)	Mean 46.3 ± 4.1	RYGB	1/13	Median 36 [23–64]	Median 28.8 [24.0–34.4]	6	3	4	1 (Pneumonia) 7 (UTI) 2 (CMV reactivation) 1 (Wound infection) 2 (Wound dehiscence) 1 (Lymphocele)	0
Bouchard et al. [49], 2020	RCoS	Canada	32	14 ( NR)	NR	SG	NR	Median 9 (IQ 32)	Median: 31 (IQ 9)	2	0	0	NR	0
Cohen et al. [50], 2019	RCoS	United States	43	38 (23/15)	Mean 49	NR	16/22	Median 43 (IQ 20–89)	Mean 32	NR	NR	NR	NR	NR
Kim et al. [21], 2020	RCoS	United States	41	41 (22/16)	Mean 56 ± 9.3	SG	16/25	Mean 21.8 ± 20.3	Mean 32.4 ± 3.4	0	1	1	8 (Wound infection) 3 (CMV reactivation)	0
Yemini et al. [51], 2019	RCoS	Israel	24	16 (13/3)	Mean 57 (range 29–70)	3 RYGB 13 SG	11/5	Mean 24 ± 5.2	Mean 29 ± 1.3	1	2	0	NR	0
Gaillard et al. [52], 2020	RCoS	France	29	12 ( NR)	NR	SG	1/11	NR	Median 30.1 (IQ 26.9–33.3)	1	0	1	1 (UTI) 1 (Septic shock)	1 (Sepsis) 1 (Unknown)
Outmani et al. [39], 2020	Case–control	Netherland	23	23 (7/16)	55.5 (IQ 40.4–61.5)	9 RYGB 11 SG 3 AGB	18/5	Mean 62	Median: 33.6 (IQ 31.4–34.7)	0	8	6	14 (UTI) 3 (Wound infection) 2 (Lymphocele)	1 (Sepsis) 1 (Malignancy)

*CR*, case report; *CS*, case series; *RCoS*, retrospective cohort study; *RCT*, randomized clinical trial; *AGB*, adjustable gastric banding; *RYGB*, Roux-en-Y gastric bypass; *SG*, sleeve gastrectomy; *NR*, not reported; *UTI*, urinary tract infection; *CMV*, cytomegalovirus

**Table 3:** Newcastle–Ottawa scale for quality assessment of the included studies

Authors	Cohort studies								
	Selection	Ascertainment		Causality				Reporting	Score
	Patients represent the whole experience of the investigators	Adequately ascertained exposure	Adequately ascertained outcome	Other alternative causes that may explain the observation were ruled out	There was a challenge/rechallenge phenomenon	Was there a dose–response effect?	Was follow-up long enough for outcomes to occur	Are the cases described with sufficient details to allow other investigators to replicate the research?	
Marks et al. [25]	-	*	*	*	N/A	N/A	*	*	Fair
Newcombe et al. [42]	-	*	*	*	N/A	N/A	-	-	Poor
Buch et al. [43]	-	*	*	*	N/A	N/A	-	*	Fair
Alexander et al. [44]	-	*	-	*	N/A	N/A	*	-	Poor
Koshy et al. [45]	-	*	-	*	N/A	N/A	-	-	Poor
Cacciola et al. [26]	-	*	*	*	N/A	N/A	*	-	Poor
Olarte et al. [27]	-	*	*	*	N/A	N/A	*	-	Poor
Bennett et al. [20]	-	*	*	*	N/A	N/A	*	*	Fair
Ditonno et al. [28]	-	*	*	*	N/A	N/A	*	-	Poor
Karabicak et al. [29]	-	*	*	*	N/A	N/A	*	-	Poor
Marszalek et al. [46]	-	*	*	*	N/A	N/A	-	-	Poor
Curran et al. [30]	-	*	-	*	N/A	N/A	*	*	Poor
Garcia-Roca et al. [31]	-	*	*	*	N/A	N/A	*	-	Poor
Tremblay et al. [32]	-	*	-	*	N/A	N/A	*	*	Poor
Schachtner et al. [33]	-	*	*	*	N/A	N/A	*	*	Fair
Al Bahri et al. [47]	-	*	*	*	N/A	N/A	-	*	Fair
Kienzl-Wagner et al. [23]	-	*	*	*	N/A	N/A	*	*	Fair
Al Sabah and Al Haddad [48]	-	*	*	*	N/A	N/A	*	*	Fair
Liese et al. [34]	-	*	-	*	N/A	N/A	*	*	Poor
Veasey et al. [35]	-	*	*	*	N/A	N/A	*	*	Fair
Bouchard et al. [49]	-	*	*	*	N/A	N/A	*	-	Poor
Cohen et al. [50]	-	*	-	*	N/A	N/A	-	*	Poor
Kim et al. [21]	-	*	*	*	N/A	N/A	*	-	Poor
Kostakis et al. [37]	-	*	*	*	N/A	N/A	*	-	Poor
Yemeni et al. [51]	-	*	*	*	N/A	N/A	*	-	Poor
Bellini et al. [38]	-	*	*	*	N/A	N/A	*	*	Fair
Gaillard et al. [52]	-	*	*	*	N/A	N/A	-	-	Poor
Prudhomme et al. [40]	-	*	-	*	N/A	N/A	-	*	Poor
Spaggiari et al. [41]	-	*	*	*	N/A	N/A	*	*	Fair
	Case–control studies								
	Selection				Comparability	Exposure			
	Adequate case definition	Representativeness of the cases	Selection of controls	Definition of controls	Comparability of cases and controls	Ascertainment of exposure	Same method of ascertainment for cases and controls	Non-response rate	
Outmani et al. [39]	*	-	*	*	*	*	*	*	Good
Thomas et al. [36]	*	-	*	*	*	*	*	*	Good

Sixteen studies reported data from obese kidney transplant recipients without prior bariatric surgery (Table 2) [20, 25–35, 37, 38, 40, 41], all of which were cohort studies, and 14 of them were considered as high quality according to the NOS scale [20, 25, 26, 28–35, 37, 38, 40]. Thirteen studies reported outcomes of patients with bariatric surgery pre-kidney transplantation [21, 23, 42–52]. Seven were retrospective cohort studies, five of these studies were case reports [42, 43, 45, 46, 48], and three were case series [23, 44, 47]. Only two studies were considered high quality [42, 49].

### Case–Control Studies

Only two non-randomized, single-centre, case–control studies from the Netherlands [39] and the USA [36] compared post-kidney transplantation outcomes between recipients with and without prior bariatric surgery.

In one case–control study, 14 patients were transplanted following Roux-en-Y gastric bypass, and 19 recipients were used as historical controls. Apart from mean BMI at the time of transplantation, being expectedly different between patients with pre-transplantation RYGB and the controls (28.9 ± 0.7 kg/m^2^ and 38.6 ± 0.4 kg/m^2^, respectively), no significant difference in baseline characteristics were present between both groups. After 72 months of follow-up, there were no significant differences between both groups for graft loss, patient mortality, delayed graft function, wound complications, infections, and lymphocele rates. Bariatric surgery prior to kidney transplantation was associated with a higher rate of biopsy-proven acute rejection compared to those without bariatric surgery pre-transplantation (6/14 vs 3/19, *p* = 0.03) [36].

The other case–control study compared transplantation outcomes for 23 and 133 kidney transplant recipients with and without prior bariatric surgery, respectively. There were no significant differences in baseline characteristics between the patient groups, other than median BMI at the time of transplantation that was expectedly lower among recipient with prior bariatric surgery (33.8 kg/m^2^) compared to the controls (36.7 kg/m^2^) as well as the prevalence of diabetes mellitus, being 39.1% versus 60.9%, respectively (*p* = 0.043) among patients with and without bariatric surgery, respectively. After a median of 61 (range: 14–258) months post-transplantation, no significant differences were present between both groups for graft loss, patient mortality, delayed graft function, wound complications and lymphocele rates. Multivariable Cox proportional hazard analysis showed that a prior history of diabetes mellitus and pre-transplantation dialysis (but not prior bariatric surgery) were independent risk factors for all-cause mortality. Bariatric surgery was associated with greater incidence of after transplantation (odds ratio: 3.37 [1.35–8.40], *p* = 0.007); however, after propensity-score matching, there was no significant difference between both groups for urinary tract infection (odds ratio: 1.42 [0.44–4.53] *p* = 0.384) [39].

#### Patients with Severe Obesity Undergoing Kidney Transplant Without Bariatric Surgery

Data from 18 studies (14 retrospective cohort studies, two prospective cohort and two case–control studies), including a total of 2134 obese kidney transplant candidates with an age range of 20 to 78 years, were reviewed (Table 1). Of these, nine studies were from the USA, three from the UK, one from Canada, and the rest were from Europe. Information about the type of donors was available for 2081 out of 2134 transplants, with the majority being living donors (1544/2081, 74.2%), and the remainder being deceased donors (537/2081, 25.8%). No studies reported donor type (whether deceased donors were donors after cardiac death or brain death). Other donor details were not reported by the included studies.

For the primary outcomes, seventeen studies reported data on graft loss, occurring in 287 of 2091 patients (13.7%) within 5 years after transplantation. Mortality rate was also reported in 15 studies, occurring in 182 of 1988 patients (9.1%) within 5 years after transplantation.

Regarding the secondary outcomes, data for acute rejection, delayed graft function, infections, wound complications and lymphocele were available for 895 (41.9%), 1993 (93.4%), 347 (16.3%), 496 (23.2%) and 288 (13.5%) patients, respectively; and incidence rates for acute rejection, delayed graft function, infections, wound complications and lymphocele were 78/895 (8.7%), 357/1993 (17.9%), 70/347 (20.2%), 69/496 (13.91%) and 21/288 (7.29%), respectively.

#### Obese Patients Undergoing Kidney Transplant with Pre-transplant Bariatric Surgery

Fifteen studies (five case reports, three case series and seven retrospective cohort studies) were reviewed. A total of 148 obese patients, aged 25 to 70 years, underwent pre-kidney transplantation bariatric surgery. Of the transplant donors, 95 were deceased (59%) and the remainder were living (41%). Sleeve gastrectomy, Roux-en-Y gastric bypass and adjustable gastric banding were used for 99 patients [21, 23, 39, 48, 49, 51, 52], 39 patients [36, 39, 44, 47, 51] and 10 patients [39, 42, 43, 45, 46], respectively. One study including 38 patients did not report the type of bariatric surgery employed [50]. BMI loss for patients with severe obesity after bariatric surgery is indicated in Table 4.

**Table 4:** Body mass index loss for patients with severe obesity after bariatric surgery

Authors	Transplanted patients, n	Months from bariatric surgery to transplant		BMI loss, kg/m^2^	BMI at transplantation, kg/m^2^
Newcombe et al. [42]	1	9		11.2	33.2
Buch et al. [43]	1	24		NR	NR
Alexander et al. [44]	9	28		NR	NR
Koshy et al. [45]	3	M#1	15	4.7	30.4
		M#2	15	6.8	36
		F#1	14	7.1	37
Marszalek et al. [46]	1	10		12.5	29
Al Bahri et al. [47]	4	F#1	54	27	NR
		F#2	66	14	
		F#3	30	20	
		M#1	63	29	
Kienzl-Wagner et al. [23]	7	F#1	23	7.8	35.7
		F#2	6	7.6	29.8
		M#1	34	6.5	28.1
		F#3	22	10.7	35.2
		M#2	7	5.2	30.3
		F#4	22	6.5	30.7
		M#3	9	6.9	29.2
Al Sabah and Al Haddad [48]	1	17		6-month: 10.5 kg/m^2^ 11-month: 8.5 kg/m^2^	31.5
Thomas et al. [36]	14	Median: 33		13.6 ± 0.8	28.9 ± 0.7
Bouchard et al. [49]	14	Median: 8 (IQ 12)		Median: 31	Median: 31 (IQ 9)
Cohen et al. [50]	38	Median: 72 (IQ 24–96)		NR	32
Kim et al. [21]	41	21.3 ± 16.4		12-month: 32.1 ± 4.7	32.4 ± 3.4
Yemeni et al. [51]	16	Median: 18 (range 1–52)		NR	29 ± 1.3
Gaillard et al. [52]	12	Median: 24 (IQ 15–31)		NR	Median: 30.1 (IQ 26.9–33.3)
Outmani et al. [39]	23	Median: 32.7 (IQ 17.2–65.2)		Median 12-month: 33.9 (IQ 31.2–36.5)	Median: 33.6 (IQ 31.4–34.7)

The duration of follow-up after transplantation ranged from 1 month to over 5 years. With respect to primary outcomes, thirteen studies (87%) reported graft loss in 12 of 138 patients (8.7%). Fourteen studies (93%) reported mortality in four of 147 patients (2.8%) (Table 2).

Data for acute rejection, delayed graft function, infections, wound complications and lymphocele were available for 138 (85.7%), 138 (85.7%), 91 (56.5%), 99 (61.5%) and 44 (27.3%) patients, respectively; and incidence rates for acute rejection, delayed graft function, infections, wound complications and lymphocele were 10/138 (7.2%), 17/138 (12.3%), 35/91 (38.5%), 16/99 (16.16%) and 4/44 (9.09%), respectively (Table 2) .

## Discussion

We aimed to review the effect of bariatric surgery in obese patients undergoing kidney transplantation compared to obese patients undergoing kidney transplantation without bariatric surgery pre-transplantation. Direct comparison between both groups was not possible as only two case–control studies directly compared outcomes between obese patients receiving bariatric surgery pre-transplantation against patients without bariatric surgery [36, 39], with the remaining studies being case reports and case series. In both these studies, no significant differences were present between groups for our primary and secondary outcomes [36, 39].

There are also many other factors than can influence the long-term post-kidney transplantation graft loss and recipient’s mortality. Recipient’s age at transplantation [53], cardiovascular complications [54] and the longer time spent on dialysis [55] are well known to be associated with poorer long-term outcome. Besides, ethnic background has been considered for many years to have an independent impact on the outcomes, as African American background has presented the lowest graft survival rate [56, 57]. The deleterious effect of recipient’s baseline metabolic diseases and the recurrence of native kidney disease are also the major causes of graft loss in long term, associated with a wide range of prognoses with different diseases [58–60]. Among factors related to the donor, deceased versus living donor, donor’s age, precise pre-transplantation evaluation of kidney donor function and longer ischemia time are established factors that influence long-term outcomes following kidney transplantation [61].

Wound complications are among the most frequently reported problems in obese kidney transplant recipients [62]. According to two case–control studies in this review, the overall reported rate of wound complications in both obese kidney transplant recipients with or without prior bariatric surgery (22/189 [11.6%]) is higher compared to the rate of 7% reported in the general kidney transplant population. However, the incidence of wound complications was similar between recipients with and those without prior bariatric surgery (3/23 [13.0%] and 3/14 [21.4%] vs 15/133 [11.3%] and 1/19 [5.3%]) [36, 39]. Nonetheless, due to the limited number of patients, we cannot make a definite statement on any association between performing bariatric surgery and severe obesity and the risk of wound complications after transplantation.

Lymphocele is also a common complication after kidney transplantation, which most frequently occurs in the first 12 postoperative weeks [63]. Several donor-related and recipient-related risk factors have been identified for lymphocele formation following kidney transplantation, including recipient obesity and diabetes mellitus [63]. While we did not find any difference in the rate of lymphocele formation after transplantation between patients with and without bariatric surgery, no studies have yet investigated whether significant pre-transplantation weight reduction alters the risk of lymphocele formation.

In the study by Outmani et al., while bariatric surgery before transplantation was associated with greater incidence of urinary tract infection post-kidney transplant, the underlying reason for this observation cannot be easily explained since the prevalence of diabetes mellitus, as a strong risk factor for bacterial infections, was greater among the bariatric surgery group, and after propensity-score matching, there was not any difference between the two groups regarding post-transplantation urinary tract infection [39]. Similarly, a recent report from the USA did not find an increased risk of various bacterial, fungal or viral infections with bariatric surgery before kidney transplantation, after controlling for baseline demographics differences, and recommended that routine infection prophylaxis after kidney transplantation to be unnecessary for patients who had undergone bariatric surgery [64].

There are several limitations to this review. The overall level of evidence among the included studies was low, because of many studies being case reports or case series with no comparator group included, selection bias, due to only non-randomized selection of the healthiest patients with severe obesity for kidney transplantation, and reporting bias, due to different lengths of follow-up and not reporting primary or secondary outcomes in most studies. Hence, due to the lack of a comparator group in all but two of the included studies, we were not able to perform meta-analysis or stratified analysis by the type of bariatric surgery or the type of donor type; due to different follow-up durations, we also were not able to define patient and graft survival at exact time points; and due to limited sample size in the two non-randomized cohort studies, we were not able to provide strong evidence on the comparison of rare complications such as mortality between the two patient groups [36, 39]. We were not able to compare patients BMI before bariatric surgery and at the time of transplantation, since relevant data were not available from many studies, the way of reporting weight loss measures were not consistent between studies, or the durations from bariatric surgery up to the time of transplantation were varied even but between patients of the same study. Similarly, we were unable to adjust for confounders since data for potential confounding factors were not available from most studies. Finally, publication bias may exist, as only English-language articles were included. The beneficial impact of bariatric surgery on the overall and cause-specific mortality following kidney transplantation among recipients with severe obesity remains an open question for future investigations, and randomized clinical trials with long enough follow-up periods are required in this regard.

## Conclusion

Data regarding post-transplantation outcomes between morbidly obese patients with and without pre-kidney transplantation bariatric surgery suggests no differences with respect to graft loss, patient mortality, delayed graft function, wound complications and lymphocele. However, this data is based on low quality studies. Randomized clinical trials directly comparing post-transplantation outcomes between patients with and without prior bariatric surgery with long enough follow-up periods are still required.

## Supplementary Information

Below is the link to the electronic supplementary material.

Supplementary file1 (DOCX 22 KB)

Supplementary file2 (DOCX 19 KB)
