# Supplementary material for: Comparison of Kidney Transplantation Outcomes Between Patients with and Without Pre-transplantation Bariatric Surgery: a Systematic Review
Source: Obes Surg. 2022 Oct 13;32(12):4066–81. doi: 10.1007/s11695-022-06308-1 (PMC9671992; doi:10.1007/s11695-022-06308-1)
Supplement: Supplementary file 1 — Supplementary file1 (DOCX 22 KB) [file 11695_2022_6308_MOESM1_ESM.docx]

| Supplementary Table 1. Selection criteria | | |
| --- | --- | --- |
|  | Inclusion | Exclusion |
| Participants | Adult patients (above 18 years) with baseline severe obesity^a^ and end-stage kidney disease who underwent kidney transplantation | Participants without severe obesity  Patients who had post-transplantation bariatric surgery  Patients who had simultaneous bariatric surgery and kidney transplantation |
| Interventions | Any type of bariatric surgery procedure performed prior to kidney transplantation | Any weight loss interventions except bariatric surgery |
| Control | Patients with severe obesity who underwent kidney transplantation without any prior attempt for weight loss | Simultaneous combined transplantations |
| Outcomes | Must include at least one of the following outcomes:   1. Acute rejection 2. Delayed graft function 3. Graft loss 4. Complications 5. Mortality | Lack of post-kidney transplantation follow-up data |
| Studies | Only full-text articles with original data were included.  Only papers written in English were included.  No restrictions were applied in the study design and the years of publication. | Grey literature, conference proceedings, book chapters, editorials, commentaries and reviews were excluded.  To avoid the risk of using duplicated data, such that the same patient would be included in at least two studies, data from the larger study was included. |
| ^a^ Severe obesity was defined as body mass index ≥35 kg/m^2^. | | |
