# Supplementary material for: Comparison of Kidney Transplantation Outcomes Between Patients with and Without Pre-transplantation Bariatric Surgery: a Systematic Review
Source: Obes Surg. 2022 Oct 13;32(12):4066–81. doi: 10.1007/s11695-022-06308-1 (PMC9671992; doi:10.1007/s11695-022-06308-1)
Supplement: Supplementary file 2 — Supplementary file2 (DOCX 19 KB) [file 11695_2022_6308_MOESM2_ESM.docx]

|  | **Search terms** |
| --- | --- |
| 1 | exp Chronic Disease/ |
| 2 | (chronic adj3 (illness* or disease* or condition*)).mp. |
| 3 | chronic disease therapy.mp. |
| 4 | or/1-3 |
| 5 | kidney diseases/ or anuria/ or diabetic nephropathies/ or hypertension, renal/ or hypertension, renovascular/ or renal insufficiency, chronic/ |
| 6 | chronic kidney disease.mp. |
| 7 | (chronic kidney or chronic renal).mp. |
| 8 | (CKD or CRD).mp. |
| 9 | Transplantations/ or Kidney/ or Transplantation/ or Kidney/ or Transplantations/ or Renal/ or Transplantation/ or Renal/ or Kidney Transplantations/ or Grafting/ or Kidney/ or Renal Transplantation/ or Kidney Grafting/ or Renal Transplantation/ |
| 10 | or/5-9 |
| 11 | Bariatric Surgery/ or Gastric Bypass/ or Gastroplasty/ or Jejunoileal Bypass/ or Lipectomy/ |
| 12 | Morbid Obesities/ or Obesities/ or Severe/ or Obesities/ or Morbid/ or Morbid Obesity/ or Obesity/ or Severe/ or Severe Obesities/ or Severe Obesity/ |
| 13 | or/11-12 |
| 14 | 4 and 10 and 13 |
| 15 | limit 14 to (english language and humans) |
